# Effect of Perioperative Benzodiazepine Use on Postoperative Delirium in Patients Undergoing Cardiac Surgery: A Systematic Review and Meta‐Analysis

**DOI:** 10.1155/anrp/5856805

**Published:** 2026-08-30

**Authors:** Marcela da Silva Kazitani Cunha, Hilária Saugo Faria, Lucas Mendes Barbosa, Leonardo Reischoffer do Nascimento, Carlos Henrique de Oliveira Ferreira, Karlos Daniell Araújo dos Santos, Lucas Cael Azevedo Ramos Bendaham, Acza Kalica Buarque da Silva, Altair Pereira de Melo Neto, Wellgner Fernandes Oliveira Amador, Ivo Queiroz Costa Neto, Ricardo Esper Treml, Caio Augusto Perete

**Affiliations:** ^1^ Faculty of Medicine of Marilia, Marilia, Sao Paulo, Brazil; ^2^ Federal University of Santa Maria, Santa Maria, Rio Grande do Sul, Brazil, ufsm.br; ^3^ Federal University of Minas Gerais, Belo Horizonte, Minas Gerais, Brazil, ufmg.br; ^4^ Amazonas State University, Manaus, Amazonas, Brazil, uea.edu.br; ^5^ Federal University of Paraiba, Joao Pessoa, Paraiba, Brazil, ufpb.br; ^6^ Federal University of Roraima, Boa Vista, Roraima, Brazil; ^7^ Federal University of Alagoas, Maceio, Alagoas, Brazil, ufal.edu.br; ^8^ Federal University of Campina Grande, Cajazeiras, Paraiba, Brazil, ufcg.edu.br; ^9^ University of Wisconsin-Madison, Madison, Wisconsin, USA, wisc.edu; ^10^ Department of Anesthesiology, Stanford School of Medicine, Stanford, California, USA, stanford.edu; ^11^ Department of Anesthesiology, Albert Einstein Israelite Hospital, Sao Paulo, Sao Paulo, Brazil, einstein.br

**Keywords:** benzodiazepine, cardiac surgery, meta-analysis, postoperative delirium

## Abstract

**Introduction:**

Cardiac surgery anesthesia is associated with risks such as postoperative delirium (POD) and stress‐related responses. Benzodiazepines have been used perioperatively to reduce anxiety and promote hemodynamic control. However, their use may increase the risk of delirium, particularly in older adults. This systematic review and meta‐analysis aims to evaluate the impact of perioperative benzodiazepine use on the incidence of POD in patients undergoing cardiac surgery.

**Methods:**

We systematically searched for randomized controlled trials (RCTs) and observational studies comparing benzodiazepines with placebo or nonbenzodiazepine drugs in adults undergoing cardiac surgery. The primary outcome was POD incidence; secondary outcomes included mini‐mental state examination (MMSE), ICU length of stay, and time to extubation. For dichotomous outcomes, odds ratios (ORs) with 95% confidence intervals (CIs) were calculated, while mean differences (MDs) were used for continuous outcomes. Analyses were performed using R 4.4.3.

**Results:**

Eight studies were included, comprising 36,301 patients, of whom 20,747 (68.5%) received benzodiazepines. Follow‐up ranged from 1 day to 12 months. There were no significant differences between groups in the incidence of POD (OR 1.05; 95% CI: 0.88 to 1.25; *p* = 0.55; I^2^ = 83%). Similarly, there were no significant difference in MMSE score (MD ‐0.11; 95% CI: −3.17 to 2.95; *p* = 0.94; I^2^ = 70%), ICU length of stay (MD 4.72 h; 95% CI: −1.37 to 10.80; *p* = 0.13; I^2^ = 99%), and time to extubation (MD ‐0.53 h; 95% CI: −2.16 to 1.09; *p* = 0.52; I^2^ = 72%).

**Conclusion:**

Perioperative benzodiazepine use was not significantly associated with POD or with secondary outcomes in adults undergoing cardiac surgery.

## 1. Introduction

Cardiac anesthesia is characterized by complex procedures, high‐risk patient profiles, maintenance of unconsciousness, and precise hemodynamic control [1]. More than one million cardiac surgeries are performed annually worldwide, and inadequate levels of hypnosis and sedation may lead to anesthesia‐related complications such as intraoperative awareness, delirium, neuroendocrine stress responses, and hemodynamic instability [2].

Cardiac surgery often induces significant anxiety, which is further intensified by invasive preanesthetic procedures and may trigger sympathetic overactivation and hemodynamic instability [3]. In this context, benzodiazepines are commonly administered for their ability to enhance GABA‐A receptor. Although recent guidelines discourage their routine perioperative use—particularly in older adults—due to concerns about postoperative delirium (POD), current meta‐analytic evidence has not demonstrated a statistically significant association between benzodiazepine use and increased risk of delirium compared to placebo or alternative agents [4, 5]. This discrepancy between clinical recommendations and emerging evidence supports continued investigation into the role of benzodiazepines in cardiac surgery, given their potential to modulate stress responses and improve patient comfort.

In light of this, we aim to perform a systematic review and meta‐analysis in order to evaluate whether benzodiazepine administration affects the incidence of delirium in adult patients undergoing cardiac surgery under general anesthesia.

## 2. Methods

This systematic review with meta‐analysis was registered in the International Prospective Register of Systematic Reviews (PROSPERO) under protocol number CRD420251034398. The registration includes the predefined research question, eligibility criteria, outcomes, and planned methodological approach. All analyses conducted in the present study strictly followed the protocol described in the PROSPERO registration, and no post hoc modifications to the analysis plan were performed. It was designed and conducted according to the Preferred Reporting Items for Systematic Reviews and Meta‐analyses (PRISMA) reporting guidelines.

### 2.1. Study Eligibility

Inclusion in this meta‐analysis was restricted to studies that met all the following eligibility criteria: (1) randomized controlled trial (RCT) or observational studies; (2) participants comprised of adults undergoing cardiac surgery; (3) studies comparing any benzodiazepine to a placebo or another nonbenzodiazepine medication; (4) studies available in full‐text, regardless of language; and (5) studies that reported at least one of the clinical outcomes of interest. We excluded studies that (1) did not report any relevant outcome and (2) involved overlapping populations.

### 2.2. Search Strategy and Data Extraction

We systematically searched PubMed, Embase, and Cochrane Central Register of Controlled Trials from inception to April 2025 with a predefined search strategy (Supporting Table 1). In addition, the references of included studies, reviews, and meta‐analyses were evaluated for additional studies. Three authors independently extracted the data, following predefined search criteria and quality assessment. Disagreements were resolved by consensus.

### 2.3. Exposure Definition and Dose Classification

We performed a structured extraction and categorization of benzodiazepine exposure across studies to account for the substantial heterogeneity in perioperative administration strategies. Exposure patterns ranged from a single preoperative oral dose to repeated boluses or continuous intraoperative infusions during cardiopulmonary bypass.

To synthesize these data, we created a table summarizing dosing strategies and administration patterns across all studies (Table 1). We categorized benzodiazepine use into the following operational groups (see Table 2):

 Preoperative (oral or sublingual dose before induction);
 Intraoperative (single IV bolus to induce anesthesia or continuous infusions during the procedure);
 Postoperative (benzodiazepines administered after intensive care unit [ICU] admission).

**TABLE 1:** Exposure definition and dose classification.

Study	Year	Country	Doses of benzodiazepine	Period of benzodiazepine administration	Number of administrations
Karski	2001	Canada	Lorazepam 0.5–2 mg SL Midazolam 0.05–0.075 mg/kg IV Midazolam intravenous infusion at 0.01 + 0.05 mg/kg/h for 3 h	Preoperative + Intraoperative + Postoperative	3 times

Imran	2021	Pakistan	Midazolam 0.05 mg/kg IV Midazolam IV infusion of 0.02–0.08 mg/kg/h	Intraoperative	2 times

Sherry	1996	England	Midazolam (3–6 mg) IV Three additional doses of midazolam (5 mg each) IV	Intraoperative	4 times

Spence	2025	North America	Midazolam, a minimum dose of 0.03 mg/kg IV, based on ideal body weight (60 kg for female patients and 70 kg for male patients)	Preoperative + intraoperative + postoperative	1–3 times

Kizilkaya	2023	Turkey	Midazolam 5 mg orally Midazolam IV infusion of 0.1 mg/kg/h	Preoperative + intraoperative	2 times

Rajaei	2019	Iran	Midazolam 0.05–0.1 mg/kg IV	Intraoperative	1 time

Searle	1997	Canada	lorazepam SL and 0.15 mg/kg	Preoperative	1 time

Yoshimura	2023	Japan	Midazolam IV. Dose not specified.	Intraoperative	1 time

*Note:* mg, milligram; kg, kilogram, SL, sublingual; IV, intravenous; h, hour.

**TABLE 2:** Operational classification of benzodiazepine exposure.

Study	Kizilkaya 2023	Rajaei 2019	Searle 1997	Yoshimura 2023
Region	Turkey	Iran	Canada	Japan

Patients, *n*, I/C	25/22	21/21	20/21	10,633/5552

Male, %, I/C	76/81.8	82.6/81.8	70/66.7	61.1/60.5

Age, y, I/C	51 (16)/45 (12)^†^	55.47 (7.18)/55.39 (6.08)^†^	55.7 (8.0)/51.2 (10.0)^†^	75.12/75.22^‡^

Weight, kg, I/C	NA	NA	75.5 (14.9)/71.8 (11.3)^†^	NA

Height, cm, I/C	NA	NA	166.5 (8.5)/165.8 (8.2)^†^	NA

Body surface area, m^2^, I/C	NA	NA	NA	23.34/23.94^‡^

ASA classification	NA	NA	II and III	NA

Hyperlipidemia	NA	11 (47.8)/14 (66.7)^#^	NA	NA

Hypertension	NA	9 (39.1)/14 (60.0)^#^	NA	80/84.2^∗∗^

Diabetes	NA	11 (47.8)/12 (54.5)^#^	NA	39/34.2^∗∗^

Surgical procedures, %, I/C	CABG with extracorporeal circulation in both groups	Elective on‐pump CABG in both groups	CABG 12/13 Valvular 8/7 ASD 0/1	Emergency procedures: 26.1/28.4 CABG 37.7/33.3 Valve plasty 7.0/5.0 Valve replacement 30.8)/19.8 SSS 0.5/0.2 TAAS 24.0/41.7

CBP time, mean (SD), min I/C	NA	NA	72.2 (24.6)/63.5 (16.8)^†^	NA

Aortic cross‐clamp time, mean (SD), min I/C	692 ± 35/94 ± 37	NA	NA	NA

Intervention group	Premedication consisted of 5 mg of midazolam orally the night before surgery and 0.5 mg of atropine IM respectively before surgery. Anesthesia was induced with 1.5 μg/kg of fentanyl + continuous infusion of 0.1 mg/kg/h midazolam during extracorporeal circulation.	Midazolam was initially administered at 0.05–0.1 mg/kg intravenously in the operating room. Anesthesia was induced with fentanyl and propofol.	Premedication consisted of 2 mg lorazepam SL and 0.15 mg/kg morphine IM given 90 and 30 min respectively before the procedure. Immediately on arrival in the ICU, propofol and placebo were started at a dose of 10 μg/kg/min. Adjustment in propofol and placebo infusions by increments of 10 μg/kg/min were permitted every 10 min to maintain an appropriate level of sedation.	In the intervention group, patients received intravenous midazolam during anesthesia induction on the day of cardiac surgery. The dose was not specified in the database used.

Control group	Premedication consisted of 0.5 mg of atropine IM respectively before surgery. Anesthesia was induced with 1.5 μg/kg of fentanyl.	Dexmedetomidine (Precedex) was initially administered at 1 μg/kg intravenously in the operating room. Anesthesia was induced with fentanyl and propofol.	Premedication consisted of 2 mg lorazepam SL and 0.15 mg/kg morphine IM given 90 and 30 min respectively before the procedure. Immediately on arrival in the ICU, the midazolam and placebo infusions were started at a dose of 0.25 μg/kg/min and both were adjusted every 10 min by an increase of 0.25 μg/kg/min when needed.	No use of midazolam

Follow‐up	72 h after extubation	30 days after surgery	24 h after arrival in the ICU	72 h after surgery

*Note: n*: number; I/C: intervention group/control group; y: years; m^2^: square meters; kg: kilogram; mg: milligram; CABG: coronary artery bypass; min: minutes; CBP: cardiopulmonary bypass.

Abbreviations: ASA, American Society of Anesthesiologists; ASD, atrial septal defect; ICU, intensive care unit; NA, not available; SD, standard deviation; SSS, supraaortic stenosis surgery; TAAS, thoracic aortic aneurysm surgery.

^#^Mean (standard deviation).

^†^Median (range).

^‡^Weighted means were calculated using the midpoint of the reported ranges, weighted by the corresponding sample size.

^∗∗^Values are expressed as percentages.

This classification was applied for descriptive purposes only, as cumulative doses and infusion durations were inconsistently reported across studies. Therefore, a dose‐response analysis was not feasible, and benzodiazepine exposure was ultimately analyzed as a binary variable (use vs. no use), following each study’s original definition.

### 2.4. Endpoints

Primary endpoint was POD, defined in three ways: (1) as an acute disturbance of the central nervous system, manifesting as disturbed consciousness, cognitive impairment, and altered perception, assessed using either the Confusion Assessment Method for the ICU (CAM‐ICU) or the Intensive Care Delirium Screening Checklist (ICDSC)—both are validated tools for the systematic detection of delirium in critically ill patients. The CAM‐ICU identifies delirium through features such as acute onset or fluctuating course, inattention, disorganized thinking, and altered level of consciousness, while the ICDSC consists of an 8‐item checklist based on DSM criteria, evaluating the presence of characteristic symptoms over a defined clinical observation period; (2) the requirement for antipsychotic medication; (3) evaluation using the scale mini‐mental state examination (MMSE) score, ranging from 1 to 30, where a patient with a score of less than 24 indicates cognitive impairment or delirium. Secondary endpoints included the ICU length of stay, time to extubation, and MMSE score.

### 2.5. Quality Assessment

Two independent authors employed the Cochrane Risk of Bias tools to evaluate the quality of the studies: ROBINS‐I for nonrandomized trials and RoB‐2 for RCTs. The risk of bias plots were created with the risk‐of‐bias visualization (ROBVIS) tool. Any disagreements were resolved through the publication bias, and Egger’s test was not assessed due to the low number of studies.

### 2.6. Statistical Analysis

We employed the inverse variance random‐effects model with a restricted maximum likelihood variance estimator for all outcomes. We pooled odds ratios (OR) for the binary outcomes and mean differences (MDss) for the continuous endpoints with 95% confidence intervals (CI). Heterogeneity was assessed using Cochran’s Q and I^2^ statistics, with *p* ≤ 0.10 indicating significance. I^2^ values were categorized as 0% (no heterogeneity), ≤ 25% (low), ≤ 50% (moderate), and > 50% (substantial). Additionally, a leave‐one‐out analysis was performed for high‐heterogeneity outcomes to evaluate the influence of each study on heterogeneity and pooled effect size. All statistical analyses were performed using R Version 4.4.3.

## 3. Results

### 3.1. Study Selection and Baseline Characteristics

**FIGURE 1:**
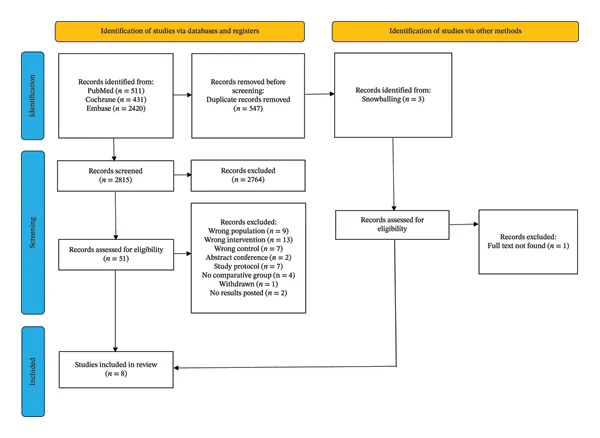
PRISMA 2020 flow diagram illustrating the selection process of studies included in the systematic review.

As detailed in Figure 1, our search retrieved 3362 records from PubMed, Embase, and Cochrane Central. After removing duplicates and screening titles and abstracts, 53 full‐text articles were assessed for eligibility, ultimately including eight studies in the final analysis. Among these, six [6–11] were RCTs, and two [12, 13] were observational studies, comprising a total of 36,301 patients, of whom 20,747 (57.2%) received benzodiazepines. The baseline characteristics of the included studies are summarized in Table 3.

**TABLE 3:** Baseline characteristics of included studies.

Study	Karski 2001	Imran 2021	Sherry 1996	Spence 2025
Region	Canada	Pakistan	England	North America

Patients, *n*, I/C	39/39	35/35	33/37	9941/9827

Male, %, I/C	76.9/66.7	NA	90.6/72.2	73.3/73.7

Age, y, I/C	70.4 (3)/71.2 (3)^#^	63.1/62.57^‡^	62 (39–77)/58 (43–81)^†^	64.9 (11.8)/65.0 (11.7)^#^

Weight, kg, I/C	77.6 (8)/78.3 (9)^#^	NA	78 (58–107)/75 (50–100)^†^	NA

Height, cm, I/C	NA	NA	NA	NA

Body surface area, m^2^, I/C	1.89 (0.24)/1.78 (0.19)^#^	NA	1.9 (1.6–2.2)/1.9 (1.5–2.2)^†^	NA

ASA classification	NA	I OR II	NA	NA

Hyperlipidemia	NA	NA	NA	NA

Hypertension	66/65	NA	NA	NA

Diabetes mellitus	30.6/32.5	NA	NA	NA

Surgical procedures, %, I/C	CABG surgery in both groups	CABG and valve or combined surgery with Cardiopulmonary Bypass in both groups	CABG 59/60 Valve replacement 24/25	Emergency procedure: 8.1/7.6 Single valve repair replacement 17.4/17.6 Isolated CABG 49.3/48.5 Other cardiac surgery 33.3/34

CBP time, mean (SD), min I/C	89.1 (22)/81.0 (20)	NA	NA	117 (58)/116 (58)

Aortic cross‐clamp time, mean (SD), min I/C	68.5 (16)/61.0 (16)	NA	NA	88 (48)/88 (47)

Intervention group	Premedication consisted of lorazepam followed by midazolam. Anesthesia was induced with fentanyl (10 ± 15 μg/kg) and midazolam (0.05 ± 0.075 mg/kg). intraoperatively and a midazolam infusion for 3 h postoperatively)	Midazolam was initially administered at 0.05 mg/kg in the operating room, followed by an intravenous infusion of 0.02–0.08 mg/kg/h to maintain anesthesia.	Premedication with morphine and hyoscine was administered intramuscularly 1 hour before surgery. Midazolam (3–6 mg) and fentanyl (60 μg/kg) were used for induction. Three additional doses of midazolam (5 mg each) were administered before CPB, at the start of rewarming, and upon termination of CPB.	A minimum dose of 0.03 mg/kg of midazolam, based on ideal body weight (60 kg for female patients and 70 kg for male patients), was administered.

Control group	Fentanyl (10 ± 15 μg/kg) and propofol (2 ± 6 mg/kg) were administered intraoperatively, followed by a continuous propofol infusion for 3 h postoperatively.	A dexmedetomidine infusion was initiated in the operating room at a dose of 0.7 μg/kg/h and was continued in the high dependency unit at a reduced dose of 0.4 μg/kg/h.	Premedication with morphine and hyoscine was administered intramuscularly 1 hour before surgery. An infusion of propofol was used for anesthesia maintenance, along with fentanyl at a dose of 15 μg/kg.	No benzodiazepine use

Follow‐up	6–12 months (telephone follow‐up for functional status)	72 h after surgery	< 1 day (until ICU discharge)	72 h after surgery

*Note: n*: number; I/C: intervention group/control group; y: years; m^2^: square meters; kg: kilogram; mg: milligram; CABG: coronary artery bypass; min: minutes; CBP: cardiopulmonary bypass.

Abbreviations: ASA, American Society of Anesthesiologists; ASD, atrial septal defect; ICU, intensive care unit; NA, not available; SD, standard deviation; SSS, supraaortic stenosis surgery; TAAS, thoracic aortic aneurysm surgery.

^#^Mean (standard deviation).

^†^Median (range).

^‡^Weighted means were calculated using the midpoint of the reported ranges, weighted by the corresponding sample size.

The mean age of participants ranged from 45 to 75 years, with hypertension and diabetes mellitus being the most frequently reported comorbidities. When described, ASA classifications ranged from II to III. The most common procedure performed was coronary artery bypass grafting (CABG), although some studies included valve and aortic surgeries [7–9, 11, 12]. Cardiopulmonary bypass was used in nearly all cases, with reported aortic cross‐clamp times ranging from 61 to 94 min. Most studies assessed outcomes within 24–72 h postoperatively, while one study included follow‐up extending up to 12 months [10]. Across the included studies, the reported range of incidence of POD varied from 8.57% to 23.9%.

### 3.2. Pooled Analysis of All Included Studies

**FIGURE 2:**
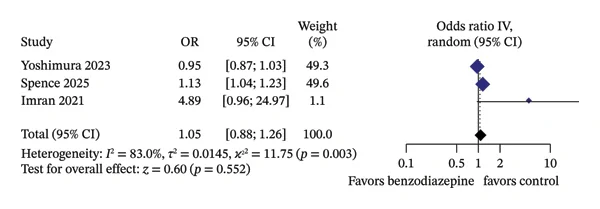
There were no significant differences between groups in the incidence of postoperative delirium (POD).

**FIGURE 3:**
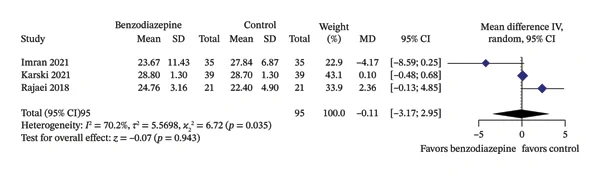
There were no significant differences between groups in the mini mental state examination (MMSE) score.

**FIGURE 4:**
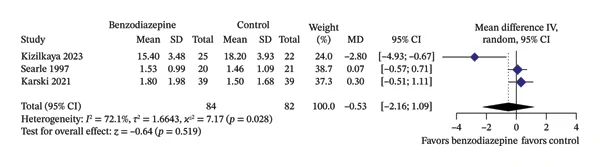
There were no significant differences between groups in time to extubation.

**FIGURE 5:**
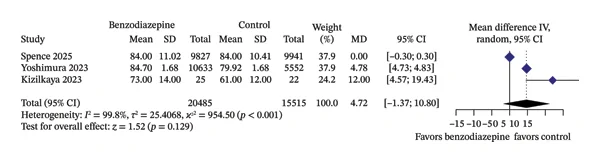
There were no significant differences between groups in the ICU Length of S.tay.

There were no significant differences between groups in the incidence of POD (OR 1.05; 95% CI: 0.88 to 1.25; *p* = 0.55; I^2^ = 83%; Figure 2). Additionally, our study found no significant difference between groups in the MMSE score (MD ‐0.11; 95% CI: −3.17 to 2.95; *p* = 0.94; I^2^ = 70%; Figure 3), time to extubation (MD ‐0.53 h; 95% CI: −2.16 to 1.09; *p* = 0.52; I^2^ = 72%; Figure 4), and ICU LOS (MD 4.72 h; 95% CI: −1.37 to 10.80; *p* = 0.13; I^2^ = 99%; Figure 5) between groups.

In the leave‐one‐out sensitivity analysis for time to extubation, excluding the Kizilkaya study reduced heterogeneity to 0% (I^2^ = 0) while preserving the pooled effect (MD 0.16; 95% CI: −0.34 to 0.66; Supporting Figure 1). For ICU LOS, omitting the Spence study resulted in a significant reduction favoring the control group (MD −7.39 h; 95% CI: −14.19 to −0.59; Supporting Figure 2). The remaining leave‐one‐out sensitivity analyses were consistent with the main findings, as shown in Supporting Figures 3 and 4.

### 3.3. Quality Assessment

The individual RCT appraisal is reported in Supporting Figure 5. Two studies were assessed as having a low risk of bias across all domains [6–11]. In contrast, three studies were evaluated as having a high overall risk of bias, primarily due to issues related to the randomization process and outcome measurement [7, 9, 10]. Additionally, one study raised some concerns regarding randomization, deviations from intended interventions, and outcome measurement [8]. The appraisal of individual observational studies is presented in Supporting Figure 6. One trial was classified as having a critical overall risk of bias, stemming from significant concerns about outcome measurement and confounding factors [12]. The other study exhibited a moderate risk due to concerns about issues in outcome measurement and reporting bias [13].

## 4. Discussion

In this systematic review and meta‐analysis including over 36,000 patients undergoing cardiac surgery, perioperative benzodiazepine administration was not significantly associated with POD. Similarly, no relevant differences were observed for secondary outcomes, including cognitive function (MMSE scores), ICU length of stay, and time to extubation.

POD is an acute neurocognitive disorder marked by fluctuating disturbances, and it is a common complication in elderly patients undergoing cardiac surgery [14]. The large cluster‐randomized crossover trial by Spence et al. [11], which enrolled 18,768 patients, demonstrated comparable delirium rates between liberal and restrictive benzodiazepine strategies during cardiac anesthesia. Similarly, Yoshimura et al. [12] analyzed 16,185 patients and reported no significant association between intraoperative midazolam use and POD in a nationwide retrospective cohort of cardiac surgical patients. Traditionally, benzodiazepines have been implicated in delirium pathophysiology due to their potentiation of GABAergic transmission, which can suppress cortical activity, disrupt sleep–wake cycles, and impair cholinergic function—mechanisms believed to contribute to the development of POD in vulnerable individuals [15]. Yet, neither of these large studies demonstrated a direct association between intraoperative benzodiazepine use and increased delirium rates. Our meta‐analysis supports these findings, suggesting that intraoperative benzodiazepine administration does not significantly influence the incidence of POD in older cardiac surgery patients. Despite this, current ERAS and geriatric perioperative guidelines still discourage routine benzodiazepine use in older adults due to concerns regarding delirium risk. Therefore, our findings should not be interpreted as sufficient evidence to reverse these recommendations, but rather as suggesting that benzodiazepines may not exert a large or clearly detectable effect on delirium incidence in contemporary cardiac anesthesia practice.

ICU length of stay is a critical postoperative outcome in cardiac surgery, as prolonged stays are associated with increased morbidity, higher healthcare costs, and poorer long‐term survival [16–18]. Kapadohos et al. identified factors such as low preoperative hemoglobin, prolonged aortic cross‐clamp time, and early postoperative hypoxemia as predictors of extended ICU stays, emphasizing the need for targeted perioperative management to optimize patient outcomes [19]. Our findings demonstrated that there was no significant difference between the groups in ICU length of stay. However, leave‐one‐out sensitivity analysis revealed that excluding the study by Spence et al. [11] resulted in a statistically significant reduction favoring the control group, along with a substantial decrease in heterogeneity. Although both Spence et al. [11] and Yoshimura et al. [12] contributed with large sample sizes, Spence’s minor effect and its cluster‐randomized design, focused on institutional policy rather than individual interventions, may have introduced systematic differences that diluted the overall effect.

Additionally, our study found no significant difference between groups in MMSE score and time to extubation. These findings suggest that perioperative benzodiazepine use may have limited impact on immediate postoperative cognitive performance, hemodynamic parameters, or ventilatory recovery when assessed in isolation. Tools such as the MMSE, while commonly used, may lack sensitivity for detecting subtle postoperative cognitive changes. As a brief screening instrument, the MMSE is not designed to detect mild neurocognitive impairment and may therefore fail to identify more subtle forms of postoperative cognitive dysfunction (POCD), which require more detailed neuropsychological testing [20, 21]. Notably, in the leave‐one‐out sensitivity analysis for time to extubation, exclusion of the Kizilkaya study markedly reduced heterogeneity (I^2^ = 0%) without affecting the pooled effect size. This is most likely explained by the unusually prolonged extubation times reported in that trial—averaging 15–18 h in both groups—which contrast with contemporary cardiac anesthesia standards promoting extubation within 6–8 h [22]. Moreover, the use of continuous midazolam infusion during cardiopulmonary bypass, as applied in Kizilkaya’s protocol, may have contributed to delayed recovery and introduced methodological divergence from modern fast‐track recovery approaches [23, 24].

The methodological quality of the included studies should be considered when interpreting these findings. According to the RoB‐2 and ROBINS‐I assessments, three RCTs presented a high overall risk of bias, mainly due to concerns related to the randomization process and outcome measurement, and one observational study was classified as having a critical risk of bias. Although a sensitivity analysis restricted to studies with lower risk of bias was considered, the small number of eligible trials and the limited number of studies classified as low risk precluded a meaningful statistical subgroup analysis. Nevertheless, when qualitatively examining the randomized trials with low overall risk of bias, the findings were consistent with the pooled estimates. In particular, the large cluster‐randomized trial by Spence et al. [11] and the randomized study by Rajaei et al. [6] did not demonstrate an increased incidence of POD associated with benzodiazepine use. Therefore, the presence of methodological limitations and substantial heterogeneity across studies reduces the certainty of the available evidence, which may be considered very low to low, and the findings should be interpreted with caution.

Overall, this meta‐analysis of eight studies and 36,301 patients reinforces recent evidence suggesting that perioperative benzodiazepine use may not increase the risk of POD in patients undergoing cardiac surgery under general anesthesia. Similarly, a prior meta‐analysis by Wang et al., which included 7663 patients undergoing various types of surgery, found no significant difference between benzodiazepine and nonbenzodiazepine groups in POD [5]. Our analysis extends this evidence by focusing specifically on cardiac surgery patients—an often underrepresented group in previous syntheses—providing targeted insight for this population. Given the frequent use of benzodiazepines in clinical practice for anxiolysis and amnesia, particularly in high‐stress procedures like cardiac surgery, these results support their cautious and selective use without necessarily increasing delirium risk [25]. Nonetheless, due to remaining variability in patient populations, dosing, and anesthetic strategies, further high‐quality research is still needed to refine clinical guidelines in this area.

### 4.1. Limitations

This study has several important limitations. First, there was variability in design and perioperative protocols across the included studies, which may have influenced the pooled estimates. To mitigate this limitation, we conducted multiple sensitivity analyses to evaluate the stability of the overall results. Second, publication bias is still a concern; however, we were not able to address this matter given the lower number of studies. Third, variations in the administration of preanesthetic medications across studies may have affected baseline sedation levels, potentially contributing to differences in outcomes. Lastly, we were unable to perform a subgroup analysis comparing intervention and placebo due to an insufficient number of studies meeting this criterion for reliable statistical analysis.

## 5. Conclusion

In this systematic review and meta‐analysis of eight studies comprising 36,301 patients undergoing cardiac surgery, perioperative benzodiazepine use was not significantly associated with an increased incidence of POD. Additionally, no significant differences were observed in MMSE scores, ICU length of stay, or time to extubation. Further high‐quality RCTs are warranted to better elucidate the safety profile of benzodiazepines in this clinical context.

**Nomenclature**
POD: Postoperative delirium
PROSPERO: Prospective Register of Systematic Reviews
PRISMA: Preferred Reporting Items for Systematic Reviews and Meta‐analyses
RCT: Randomized controlled trial
MMSE: Mini‐mental state examination
ICU: Intensive care unit
RoB‐2: Risk of bias tool
ROBINS‐I: Risk of bias in nonrandomized studies of interventions
ROBVIS: Risk‐off‐bias visualization
OR: Odds ratios
MD: Mean difference
CI: Confidence interval
CABG: Coronary artery bypass graft

## Author Contributions

Marcela da Silva Kazitani Cunha: conceptualization, methodology, formal analysis, visualization, and writing–original draft. Caio Augusto Perete and Ricardo Esper Treml: conceptualization and supervision. Hilária Saugo Faria: methodology, data curation, and investigation. Lucas Mendes Barbosa: methodology and formal analysis. Leonardo Reischoffer do Nascimento: data curation and validation. Carlos Henrique de Oliveira Ferreira: data curation. Karlos Daniell Araújo dos Santos: formal analysis. Lucas Cael Azevedo Ramos Bendaham, Acza Kalica Buarque da Silva, and Altair Pereira de Melo Neto: investigation. Wellgner Fernandes Oliveira Amador: validation. Ivo Queiroz Costa Neto: software.

## Funding

The authors declare that no funds, grants, or other support were received during the preparation of this manuscript.

## Disclosure

All authors take responsibility for all aspects of the reliability and freedom from bias of the presented data and their discussed interpretation.

## Conflicts of Interest

The authors declare no conflicts of interest.

## Supporting Information

Additional supporting information can be found online in the Supporting Information section.

## Supporting information


**Supporting Information** Supporting Methods 1. PRISMA 2020 main checklist. Supporting Methods 2. PRISMA 2020 abstract checklist. Supporting Table 1. Details of the search strategy. Supporting Figure 1. Leave‐one‐out for time to extubation. Supporting Figure 2. Leave‐one‐out for ICU LOS. Supporting Figure 3. Leave‐one‐out for the incidence of postoperative delirium. Supporting Figure 4. Leave‐one‐out for MMSE. Supporting Figure 5. Critical appraisal of individual studies according to the Cochrane Collaboration’s tool for assessing risk of bias in randomized‐controlled trials. Supporting Figure 6. Critical appraisal of individual studies according to the Cochrane Collaboration’s tool for assessing risk of bias in nonrandomized controlled trials.

## Data Availability

The data supporting the findings of this study are derived from previously published articles, which are cited in the reference list. No new data were generated.
